# Effect of biochanin A on the rumen microbial community of Holstein steers consuming a high fiber diet and subjected to a subacute acidosis challenge

**DOI:** 10.1371/journal.pone.0253754

**Published:** 2021-07-21

**Authors:** Brittany E. Harlow, Michael D. Flythe, James L. Klotz, David L. Harmon, Glen E. Aiken

**Affiliations:** 1 United States Department of Agriculture, Forage Animal Production Research Unit, Agricultural Research Service, Lexington, KY, United States of America; 2 Department of Animal and Food Sciences, University of Kentucky, Lexington, KY, United States of America; 3 North Florida Research and Education Center, University of Florida, Quincy, FL, United States of America; University of Illinois, UNITED STATES

## Abstract

Subacute rumen acidosis (SARA) occurs when highly fermentable carbohydrates are introduced into the diet, decreasing pH and disturbing the microbial ecology of the rumen. Rumen amylolytic bacteria rapidly catabolize starch, fermentation acids accumulate in the rumen and reduce environmental pH. Historically, antibiotics (*e*.*g*., monensin, MON) have been used in the prevention and treatment of SARA. Biochanin A (BCA), an isoflavone produced by red clover (*Trifolium pratense*), mitigates changes associated with starch fermentation *ex vivo*. The objective of the study was to determine the effect of BCA on amylolytic bacteria and rumen pH during a SARA challenge. Twelve rumen fistulated steers were assigned to 1 of 4 treatments: HF CON (high fiber control), SARA CON, MON (200 mg d^-1^), or BCA (6 g d^-1^). The basal diet consisted of corn silage and dried distiller’s grains *ad libitum*. The study consisted of a 2-wk adaptation, a 1-wk HF period, and an 8-d SARA challenge (d 1–4: 40% corn; d 5–8: 70% cracked corn). Samples for pH and enumeration were taken on the last day of each period (4 h). Amylolytic, cellulolytic, and amino acid/peptide-fermenting bacteria (APB) were enumerated. Enumeration data were normalized by log transformation and data were analyzed by repeated measures ANOVA using the MIXED procedure of SAS. The SARA challenge increased total amylolytics and APB, but decreased pH, cellulolytics, and *in situ* DMD of hay (P < 0.05). BCA treatment counteracted the pH, microbiological, and fermentative changes associated with SARA challenge (P < 0.05). Similar results were also observed with MON (P < 0.05). These results indicate that BCA may be an effective alternative to antibiotics for mitigating SARA in cattle production systems.

## Introduction

The rumen is an anaerobic ecosystem in which nutrients that the animal consumes are fermented by a resident phylogenetically and metabolically diverse microbial community [1]. These fermentative substrates from the diet can be converted into short chain fatty acids (SCFA), which are then removed from the rumen by absorption into the blood stream or passage to the lower tract. A healthy stable rumen environment can be maintained if substrate fermentation rates are not in excess of buffering capacity (primarily via salivary secretions) and the rate of absorption/passage keeps up with production of SCFA. Subacute rumen acidosis (SARA) is commonly associated with adaptation to high grain diets. The pathogenesis of SARA is characterized by the accumulation of SCFA in the rumen leading to environmental pH decline (pH 5.6–5.0; [2–5]). When an adapted animal consumes a diet rich in starch, resident amylolytic bacteria (*e*.*g*., lactobacilli and *Streptococcus bovis*) proliferate and produce SCFA and lactic acid [6]. Rumen lactate-utilizing bacteria (*e*.*g*., *Megasphaera elsdenii*, *Selenomonas ruminatium*) then metabolize lactate into the SCFA propionate. When environmental pH drops below 5.6, SCFA absorption rate is enhanced preventing excess SCFA accumulation in the environment and further pH decline [7].

Despite this adaptation, low pH can have negative impacts on the fermentative efficiency of other rumen microbial guilds, production of microbial toxins, and animal physiology. For example, resident cellulolytic bacteria that are solely responsible for dietary fiber utilization, do not grow at a rumen pH < 6.0 leading to decreases in viable numbers and consequent fiber digestibility (*in situ* studies: 20–25% reduction in fiber digestibility; [8–10]). Additionally, rumen ciliated protozoa are extremely sensitive to reductions in rumen pH [11–13]. Subacute rumen acidosis has also been associated with damage to the rumen epithelial tissue (rumenitis), increased endotoxin and inflammatory mediators, liver abscesses, and laminitis [5,6,14–16].

Feed additive antimicrobials (*e*.*g*., monensin, virginiamycin, lasalocid) are often employed in the prevention and treatment of SARA in cattle [17–20]. Dietary inclusion of the ionophore, monensin (MON), has been demonstrated to: reduce the incidence of acidosis via antimicrobial action against amylolytic bacteria [21,22], improve feed efficiency [23,24], decrease rumen methane production [25–27], and increase rumen bypass protein by inhibiting amino acid/peptide-fermenting bacteria (APB) in the rumen [28–30]. Despite the advantages of MON, due to the concern for the spread of antibiotic resistance both the European Union [31] and the United States [32,33] have taken steps to reduce the use of antibiotics in food animal production systems. Therefore, antibiotic alternatives are of interest for the treatment and prevention of SARA. In addition to these regulatory issues, it is important to note that a marked disadvantage of commonly utilized feed antibiotics (e.g., ionophores like monensin) is that they have a broad spectrum of activity, which include some rumen cellulolytic bacteria [34]. Discovery and development of novel antimicrobials provides the opportunity to find compounds with improved spectra of activity, reduced toxicity and other advantages over currently available products.

Many plants, including forage species in pastures, can produce antimicrobial secondary metabolites that can have beneficial impacts on rumen fermentation and function [35–38]. Biochanin A (BCA), an isoflavone produced by the forage legume red clover (*Trifolium pratense*), has been shown to reverse the impacts of rumen acidosis via selective antimicrobial action in an *in vitro* and *ex vivo* model [39]. Additionally, BCA has been shown to inhibit APB [38,40–43], promote cellulolytic bacteria and fiber degradation [39,42,43], and improve average daily gains of grazing cattle [41,43]. Therefore, BCA could potentially serve as an effective mitigation strategy for SARA, but evaluation *in vivo* is required. The following experiment was conducted to determine the microbiological impacts of BCA in comparison to the feed antimicrobial MON in steers subjected to a short 2-step SARA challenge. The hypotheses were that BCA would: 1) mitigate microbiological changes and pH decline associated with the SARA challenge, 2) increase cellulolytic viable numbers and fiber degradation, and 3) suppress rumen APB.

## Materials and methods

All procedures were approved by the Institutional Animal Care and Use Committee at the University of Kentucky (protocol #: 2016–2450). General housing and care of the animals were consistent with the Guide for Care and Use of Agricultural Animals in Research and Teaching [44].

### Experimental design and diets

Twelve rumen fistualted Holstein steers (Initial BW: 373 ± 7.5 kg) with no history of antibioitic administration or gastrointestinal disease in the preceding 4 months were blocked by weight and randomly assigned to 1 of 4 treatments: 1) high fiber control (HF CON; n = 3), 2) SARA control (SARA CON; n = 3), SARA + monensin treatment (MON CON; n = 3), or SARA + BCA treatment (BCA; n = 3). The experiment was conducted over 29 days. All steers were first adapted to the basal diet (corn silage + 2.27 kg dried distillers’ grains, to meet protein requirements; Table 1) for 14 days (adaptation period). After the adaptation period, the steers remained on the basal diet for another 7 days (high fiber period). During the high fiber period, the MON and BCA treatments were mixed in loose mineral and dosed transruminally (through the cannula) with 0.45 kg of dried distillers’ grain, at 09:00 each day. The MON medicated loose mineral was dosed to provide 200 mg of MON per day as per manufacturer’s direction (Rumensin Beef Mineral Medicated Premix, KNS #634; Kentucky Nutrition Service, Lawrenceburg, KY, USA). Purified BCA (Indofine Chemical Company Inc., Hillsborough, NJ, USA) was dosed at 6 g per day (representing ~30% red clover diet; [41]) in an equal amount of conventional loose mineral (Beef Pasture Mineral, KNS #600; KNS). The high fiber control and SARA control steers also were dosed transruminally with an equal amount of dried distillers’ grain and loose mineral. Following the high fiber period, the high fiber control steers remained on the basal diet. All other steers were subjected to a cracked corn challenge designed to elicit SARA consisting of a 40% cracked corn diet (40% cracked corn + 60% basal diet, as fed basis) for 4 days (Table 1; 60:40 SARA challenge period) immediately followed by a 70% cracked corn diet (70% cracked corn + 30% basal diet, as fed basis) for 4 days (30:70 SARA challenge period). Transruminal dosing of dried distillers’ grain, loose mineral, and treatments when applicable continued during the SARA challenge periods.

**Table 1:** Nutrient composition corn silage, dried distillers’ grain (DDG), and cracked corn (dry matter basis)^1^.

Nutrient^2^	Corn Silage	DDG	Cracked Corn
**DM, %**	45.0	86.0	88.0
**CP, %**	8.39	25.18	9.77
**ADF, %**	25.75	18.62	19.21
**NDF, %**	50.11	27.65	12.27
**IVTD, %**	78.91	84.71	71.31

^1^Basal diet: Corn silage *ad libitum* + 2.27 kg DDG; 60:40 SARA Challenge Period: 60% basal diet:40% cracked corn, as fed; 30:70 → 30% basal diet:70% cracked corn, as fed.

^2^DM-dry matter; CP-crude protein; ADF-acid detergent fiber; NDF-neutral detergent fiber; IVTD–*in vitro* true digestibility.

Holstein steers were housed at the University of Kentucky C. Oran Little Research Center Beef Unit in Woodford County, KY, USA. Steers were group housed in outdoor pens for the first 7 days of the adaptation period. For the remainder of the study (22 days), steers were housed in outdoor individual pens (3 x 3.7 m) to allow for monitoring of feed intake daily. Steers were allowed 1 h of group turnout daily immediately prior to feeding (09:00) when individually housed. All steers were provided water and the basal diet ± cracked corn *ad libitum* over the course of the study. Free choice conventional loose mineral (KNS #600) was available to all steers during the adaptation period. Samples of dietary components were collected every week over the course of the experiment. All samples were dried, ground to pass through a 1-mm screen using a Wiley mill, and analyzed for CP, ADF, NDF, and true in vitro digestible DM (IVTD). Crude protein was analyzed using a Leco FP-215 N Analyzer (CP = % N × 6.25; Leco Corp., St. Joseph, MI, USA). Acid detergent fiber and NDF were analyzed using an ANKOM fiber analyzer as a modification to procedures of Goering and Van Soest ([45]; addition of α-amylase to the NDF procedure). True IVTD was determined by following procedures for estimating true digestibility using the ANKOM Daisy II Incubator [46].

### Fistulated Holstein rumen sampling

Rumen fisulated Holstein steers were sampled at 4 h post-feeding (13:00) on the last day of each experimental period (d 14, 21, 25, 29). On each sample day, rumen contents (~ 1 kg) were collected from each steer individually. Immediately post-collection, ~250 g of the contents were strained through 3 layers of cheesecloth (Grade #90) to collect rumen fluid. A portion of the collected rumen fluid (15 mL) was placed on ice and frozen for later fermentation end-product analyses (described below). The remaining collected rumen fluid was used to inoculate enumeration media (10-fold dilution, as described below) and for pH determination within 10 min of collection. Rumen fluid pH was taken with a pH meter (Accumet™ Research, AR10 pH meter; Fisher Scientific, USA with a Broadley-James® pH electrodes; Broadley® James, Irvine, CA, USA), calibrated to pH 4.0 and 7.0, at 4 h post-feeding on all sample days. Additionally, on both SARA challenge period sample days (d 25 and 29), rumen fluid pH was determined with a pH meter at 0 (immediately prior to morning feeding), 2, 4 and 8 h.

### Fistulated Holstein *In situ* Dry matter (DM) disappearance

*In situ* procedures were selected based on the recommendations for standardization by Vanzant and colleagues [47]. For the last 72 h of each experimental period, Dacron bags (10 cm × 20 cm; pore size = 50 ± 10 microns porosity; ANKOM Technology, Macedon, NY, USA) containing 5 g of finely ground (2 mm screen; model 4 Wiley Mill; Thomas Scientific, Swedesboro, NJ, USA) low-quality or high-quality grass hay (Table 2) were placed in the rumen of each animal. Both hay quality types utilized consisted of an orchardgrass and timothy mixed hay. Bags in duplicate were inserted in a weighted mesh bag in the rumen at approximately 0, 24, 48, 60, 66, and 70 h to be removed together at 72 h. Two blank Dacron bags incubated for 72 h were used to correct for rumen microbial and feed contamination. Loss of DM during rinsing was determined using two non-incubated bags for each animal. Upon removal, bags were rinsed 5 times with cold water (1min rinse^-1^) using a commercial washing machine set at low water level. Rinsed bags were dried at 55°C for 48 h, and reweighed to determine DM disappearance. Dry matter disappearance was determined for all *in situ* replicates.

**Table 2:** Nutrient composition of low- and high-quality hay (dry matter basis).

Nutrient	Low-Quality Hay	High-Quality Hay
**DM, %**	89.0	89.0
**CP, %**	7.45	11.65
**ADF, %**	43.34	36.13
**NDF, %**	70.13	59.42
**IVTD, %**	69.12	83.29

DM-dry matter; CP-crude protein; ADF-acid detergent fiber; NDF-neutral detergent fiber; IVTD–*in vitro* true digestibility.

### Media and bacterial enumerations

Immediately following collection, each rumen fluid sample was serially diluted (10-fold w/w) in anaerobic phosphate buffered saline (pH 7.4, N_2_-sparged; 8 g NaCl, 0.2 g KCl, 1.44 g Na_2_HPO_4_ per L) for the inoculation of enumeration media (described below).

The carbohydrate-utilizing bacteria basal medium used was based on Mantovani and Russell [48]. This medium was heavily buffered to maximize growth, and contained (per liter): 240 mg KH_2_PO_4_, 240 mg K_2_HPO_4_, 480 mg (NH_4_)_2_SO_4_, 480 mg NaCl, 64 mg CaCl_2_ · 2H_2_O, 100 mg MgSO_4_ · 7H_2_O, 600 mg cysteine hydrochloride, 1,000 mg Trypticase, 500 mg yeast extract; initial pH 6.7; autoclaved to remove O_2_ and cooled under O2 –free CO_2_. The buffer (4,000 mg Na_2_CO_3_) was added before dispensing and autoclaving for sterility.

The carbohydrate-utilizing bacteria medium with soluble starch from potato as a substrate (10,000 mg L^-1^) was used for enumeration of total amylolytic bacteria [49,50]. The tubes were incubated (39 **°**C, 3 d). The highest dilution exhibiting bacterial growth (visual examination, OD_600_) was recorded as the viable number.

Total cellulolytic medium was based on Stack et al. [51]. This medium consisted of carbohydrate-utilizing bacteria medium amended with 1 mg phenylacetic acid, 3.1 mL VFA solution (7.15 mol L^-1^ acetic acid,1.92 mol L^-1^ propionic acid, 783.66 mmol L^-1^ butyric acid, 222.28 mmol L^-1^ valeric acid, 216.19 mmol L^-1^ isovaleric acid, 264.13 mmol L^-1^ isobutyric acid, 227.87mmol L^-1^ 2-methylbutyric acid), and Whatman #1 filter paper strips as the growth substrate. Additionally, the viable number of 2-deoxy, D- glucose (2-DG) resistant cellulolytic bacteria were enumerated by amending the total cellulolytic medium with 2-DG (6 mmol L^-1^). Incubations were carried out at 39 **°**C for 10 d. Growth was evaluated daily by dissolution of the cellulose substrate and microscopy. The final dilution exhibiting dissolution of cellulose on d 10 was recorded as the viable number of cellulolytic bacteria.

Bile esculin azide agar (Enterococcosel™; Becton, Dickinson and Co. (BD), Franklin Lake, NJ, USA) and Rogosa SL agar (BD) were used for aerobic enumeration of Lancefield Group D Gram-positive cocci (GPC; enterococci and streptococci) and lactobacilli, respectively. Both media types were prepared in Petri plates according to the manufacturer’s directions. The plates were incubated aerobically (39 **°**C, 3 d). Plates with 30 < x < 300 colonies were counted. Black colonies on bile esculin azide agar were counted as GPC. All colonies on Rogosa SL agar were counted as lactobacilli.

The low-N basal medium contained (per liter): 240 mg KH_2_PO_4_, 240 mg K_2_HPO_4_, 480 mg Na_2_SO_4_, 480 mg NaCl, 64 mg CaCl_2_ · 2H_2_O, 100 mg MgSO_4_ · 7H_2_O, and 600 mg cysteine hydrochloride, with additional vitamins and trace minerals as previously described [52]. The initial pH was adjusted to 6.7 and the medium was autoclaved (121 **°**C, 103 kPa, 20 min) to remove O_2_ and cooled under O_2_-free CO_2_. The buffer (4,000 mg Na_2_CO_3_) was added before dispensing and autoclaving for sterility.

Total peptide-utilizing and amino acid-utilizing bacteria were enumerated in low-Nbasal media amended to include either 15 mg mL^-1^ Trypticase or 15 mg mL^-1^ Casamino acids, respectively (Becton Dickinson, Franklin Lake, NJ). Inoculated tubes were incubated (39 **°**C, 3 d), and the highest dilution exhibiting bacterial growth (visual examination, OD_600_) was recorded as the viable number.

Total gelatin-hydrolyzing bacteria were enumerated in low-N basal media amended to include 100 mg mL^-1^ gelatin (added prior to 2^nd^ autoclaving step). Tubes were inoculated and incubated (39 **°**C, 5 d), and the highest dilution exhibiting bacterial growth (visual examination of gelatin hydrolysis after 1 h at 4 **°**C) was recorded as the viable number.

### Fermentation end-product analyses by HPLC

Rumen fluid samples were thawed and clarified in a microcentrifuge (21,000 × *g*, 2 min). Short chain fatty acids (SCFA; lactate, acetate, propionate, butyrate, IVMB: isovalerate and/or methylbutyrate, and valerate) were quantified using HPLC (Summit HPLC; Dionex, Sunnyvale, CA, USA) equipped with an anion exchange column (Aminex, HP-87H; Bio-Rad, Hercules, CA, USA), refractive index (Shodex/Showa Denko, Kanagawa, Japan) and UV detector. The eluting compounds were isocratically separated with a sulfuric acid mobile phase (5 mmol L^-1^, aqueous). The column was operated at 50°C with a 0.4 mL min^-1^ flow rate and 0.1 mL injection volume.

### Statistical analyses

Prior to statistical analyses bacterial enumerations were normalized by log transformation. All data were analyzed using repeated measures in PROC MIXED of SAS, with individual steer being used as the experimental unit (SAS version 9.3, SAS Inst. Inc., Cary, NC; [53]). In the analyses block, sample day, sampling time (pH and *in situ* DM disappearance), treatment, the interaction between treatment and sample day, and the interaction between treatment and sampling time (pH and *in situ* DM disappearance) were analyzed as fixed effects with animal as the random variable. The Kenward-Roger method was used to compute the denominator degrees of freedom for the fixed effects and the repeated statement requested the compoud symmetry covariance structure. Mean separations were done using the PDIFF option of SAS. Statistical significance was set at *P* < 0.05 with trends observed at *P* < 0.10.

## Results and discussion

### Rumen pH, SCFA and amylolytic bacteria

Feed intake was unaffected by treatment and period (*P* > 0.05). Total feed intake on average was 2.9 ± 0.03% of BW on a DM basis over the course of the experiment. The average rumen fluid pH during the adaptation period, the high fiber period, and in HF CON steers over the course of the study (basal diet only) was 6.23 (range, 6.17–6.28) at 4 hours post-feeding. Steers that stayed on the basal HF diet did not display any significant rumen pH changes, either more or less acidic than the adaptation period, during any experimental period (*P* > 0.05). When steers were challenged with 40 or 70% cracked corn, pH declined and there was a treatment × time interaction for all time points (*P* ≥ 0.01, in all cases; Table 3). Rumen fluid pH in steers consuming cracked corn with no treatment (SARA CON) was ~0.5 and ~0.9 pH units lower (regardless of time point) than HF CON steers consuming the basal diet during the 60:40 and 30:70 SARA challenge periods, respectively (*P* < 0.05). BCA and MON were both effective in partially mitigating pH decline associated with either SARA challenge (*P* < 0.05). BCA treatment was found to be more effective during the 60:40 and 30:70 SARA challenge periods at 2, 4 and 8 h (excluding 2 h during the 30:70 SARA challenge period where they were equally effective) in comparison to MON (*P* < 0.05). However, despite the statistical differences observed between BCA and MON, the difference in pH mitigation between these treatments were not biologically meaningful.

**Table 3:** Effect of biochanin A on rumen fluid pH during a 40% and 70% corn SARA challenge at 0 (pre-feeding), 2, 4, and 8 h (true means; n = 3).

Hour^1^	SARA Challenge Period^2^	Treatments^3^				Statistics^4^	
		HF CON	SARA CON	MON CON	BCA	Sig	SEM
**0**	**60:40**	6.64^a^	6.16^cα^	6.39^bα^	6.48^bα^	*P* < 0.01	0.03
**0**	**30:70**	6.65^a^	5.58^cβ^	5.99^bβ^	5.94^bβ^		
**2**	**60:40**	6.02^a^	5.56^dα^	5.71^cα^	5.83^bα^	P = 0.01	0.03
**2**	**30:70**	6.09^a^	5.36^cβ^	5.61^bβ^	5.68^bβ^		
**4**	**60:40**	6.12^a^	5.49^dα^	5.76^cα^	5.92^bα^	*P* < 0.01	0.02
**4**	**30:70**	6.10^a^	5.28^dβ^	5.43^cβ^	5.59^bβ^		
**8**	**60:40**	6.22^a^	5.72^dα^	5.82^cα^	6.00^bα^	*P* < 0.01	0.03
**8**	**30:70**	6.23^a^	5.28^dβ^	5.65^cβ^	5.79^bβ^		

^1^Hour 0 is pre-feeding. Hours 2, 4 and 8 are post-feeding.

^2^60:40 → 60% basal diet:40% cracked corn; 30:70 → 30% basal diet:70% cracked corn.

^3^HF CON: High Fiber Control (basal diet only; corn silage + dried distillers’ grains, to meet protein requirements); SARA CON: SARA Control; MON CON: SARA + Monensin (200 mg d^-1^); BCA: SARA + Biochanin A (6 g d^-1^).

^4^Values with different English letters (^a,b,c,d^) are statistically different within period between treatments (*P* < 0.05); Values with different Greek letters (^α,β^) are statistically different within treatment between periods (*P* < 0.05).

It is widely accepted and has been repeatedly demonstrated that rumen pH stability is a critical factor in the maintenance of normal and stable rumen function including both microbial community (*e*.*g*., viability and fermentative capacity) and physiological stability (*e*.*g*., motility and absorptive functions; [54]). Although rumen pH will fluctuate in proximity to feed consumption, is dependent on dietary composition, and can be influenced by animal to animal variation, normal rumen pH is typically considered to be > 5.6 [5] Therefore, accumulation of SCFA and a decrease in rumen pH below 5.6 can have a significant impact on microbial community stability, overall rumen health and consequently animal productivity and health. Subacute rumen acidosis has been defined as a rumen pH ranging between 5.0 and 5.6 [2–5]. Based on this definition, all SARA CON steers in the current study suffered from SARA at 2 and 4 h post-feeding during the 60:40 SARA challenge period and at all time points tested during the 30:70 SARA challenge period. Therefore, both cracked corn challenge levels were effective at inducing SARA. No MON or BCA treated steers were in SARA during the 60:40 challenge period demonstrating that both treatments were able to mitigate SARA development at a 40% cracked corn diet concentration. In contrast, when steers were stepped up to a 70% cracked corn diet, MON treated steers entered SARA at 2 (2/3 steers), 4 (all 3 steers) and 8 h (1/3 steers) post-feeding, but BCA treated steers suffered from SARA only at 4 h (2/3 steers). The minimum amount of time of suboptimal rumen pH required to have negative impacts on microbial and physiological rumen function is unknown. However, studies utilizing continuous culture systems have suggested that there is a direct relationship between increasing duration of suboptimal pH and a decline in microbial stability and fermentative capacity [55–60]. Continuous rumen pH monitoring was not evaluated in the current study, but the interval pH data that was collected suggest that at higher levels of cracked corn supplementation (70% in the current study), BCA treatment may be more effective than MON at decreasing the duration of time that the rumen pH was suboptimal for fermentation. It is important to note that the corn challenge utilized in the current study was short in comparison to what animals would be exposed to in finishing systems (> 100 d). Therefore, although the corn challenge was effective at inducing SARA, a longer exposure may elicit different results.

When steers consumed a basal diet of corn silage and dried distillers’ grains to meet protein requirements *ad libitum*, on average rumen SCFA concentrations were approximately: 40 mmol L^-1^ acetate, 32 mmol L^-1^ propionate, 16 mmol L^-1^ butyrate, 1.6 mmol L^-1^ IVMB, 2 mmol L^-1^ valerate and 91 mmol L^-1^ total SCFA (Table 4). Although lactate was detected in some rumen fluid samples, it was negligible and below the level of quantification (< 1 mmol L^-1^). There was no individual effect or interaction of treatment and sample day on butyrate, IVMB, or valerate concentrations (P > 0.05, in all cases). A treatment × sample day interaction was detected for acetate, propionate, total SCFA and acetate to propionate ratio (P < 0.01, in all cases). Steers treated with BCA had higher rumen fluid acetate concentrations (+10 mmol L^-1^) and total SCFA (+14 mmol L^-1^) during the high fiber period than any other treatment (P < 0.05). The apparent promotion of acetate production with BCA treatment is consistent with previous results Acetate is a common product of both ecological generalist microbiota and cellulolytic bacteria [54]. Previous results from our research group indicated that BCA was selectively inhibitory to some cellulolytic bacteria, but the net impact was increased acetate concentrations and DMD by mixed rumen microbiota during *in vitro* experiments [42]. Similarly, rumen fluid from grazing steers showed greater acetate concentrations and fiber digestion when the steers were supplemented with isoflavones via red clover hay [43].

**Table 4:** Effect of biochanin A on rumen fluid short chain fatty acid (SCFA) concentrations at 4 h post-feeding (mmol L^-1^; true means; n = 3).

Treatment^1^		Experimental Period^2^			Statistics^3^	
	Adaptation	High Fiber	60:40 SARA	30:70 SARA	Sig	SEM
**Acetate**					***P* < 0.01**	**1.75**
**HF CON**	40.0	40.3^α^	40.0^β^	40.0^βγ^		
**SARA CON**	39.0^b^	40.3^α,b^	39.7^β,b^	26.3^α,a^		
**MON CON**	37.3^ab^	40.3^α,b^	33.7^α,a^	35.7^β,ab^	37.33333333	
**BCA**	40.3^a^	50.7^β,b^	47.0^γ,b^	42.7^γ,a^		
**Propionate**					***P* < 0.01**	**1.86**
**HF CON**	31.7	33.3^αβ^	30.7^α^	32.7^α^		
**SARA CON**	31.7^a^	29.7^α,a^	63.0^γ,b^	75.3^δ,c^		
**MON CON**	33.7^a^	36.3^β,a^	45.3^β,b^	55.3^γ,c^		
**BCA**	31.0^a^	36.3^β,ab^	31.0^α,a^	42.3^β,b^		
**Butyrate**					*P* = 0.29	1.72
**HF CON**	16.0	13.3	13.3	14.7		
**SARA CON**	16.7	16.0	15.0	19.3		
**MON CON**	18.7	12.3	13.7	14.3		
**BCA**	17.7	13.3	18.3	13.3		
**Total SCFA**^**4**^					***P* < 0.01**	**2.96**
**HF CON**	90.8	90.6^α^	87.9^α^	91.4^α^		
**SARA CON**	90.7^a^	90.3^α,a^	122.0^γ,b^	128.0^γ,b^		
**MON CON**	92.4^a^	93.1^α,a^	96.0^αβ,a^	109.8^β,b^		
**BCA**	92.8^a^	104.9^β,b^	100.2^β,ab^	102.4^β,b^		
**Acetate:Propionate Ratio**					***P* < 0.01**	**0.09**
**HF CON**	1.3	1.2^αβ^	1.3^β^	1.2^γ^		
**SARA CON**	1.2^c^	1.4^αβ,c^	0.6^α,b^	0.4^α,a^		
**MON CON**	1.1^b^	1.1^α,b^	0.7^α,a^	0.6^β,a^		
**BCA**	1.3^a^	1.4^β,a^	1.5^β,a^	1.0^γ,b^		

^1^HF CON: High Fiber Control (basal diet only); SARA CON: SARA Control; MON CON: SARA + Monensin (200 mg d^-1^); BCA: SARA + Biochanin A (6 g d^-1^).

^2^Adaptation: 100% basal diet (corn silage + dried distillers’ grains, to meet protein requirements); High Fiber: 100% Basal Diet; 60:40 SARA: 60% basal diet:40% cracked corn; 30:70 SARA: 30% basal diet:70% cracked corn.

^3^Values with different English letters (^a,b,c,d^) are statistically different within treatment between periods (*P* < 0.05); Values with different Greek letters (^α,β^) are statistically different within period between treatments (*P* < 0.05).

^4^Total SCFA = Acetate + Propionate + Butyrate + Valerate + IVMB (isovalerate and/or methylbutyrate).

When steers were challenged with 40% cracked corn (SARA CON), propionate (+32 mmol L^-1^) and total SCFA (+34 mmol L^-1^) concentrations increased, and consequently the acetate to propionate ratio decreased in comparison to HF CON (P < 0.05). Treatment with MON decreased propionate and acetate production in comparison to SARA CON and HF CON, respectively (P < 0.05). BCA treated steers had similar propionate concentrations (P > 0.05) but had increased acetate (+7 mmol L^-1^) and total SCFA (+12 mmol L^-1^; P < 0.05), allowing for maintenance of an acetate to propionate ratio that was present in the HF CON steers (P > 0.05). After being stepped up to a 70% cracked corn challenge, propionate (+42.6 mmol L^-1^) and total SCFA (+37 mmol L^-1^) concentrations continued to increase in SARA CON steers and acetate (-14 mmol L^-1^) decreased in comparison to HF CON (P < 0.05). Both MON and BCA treatment partially mitigated the increase in propionate and total SCFA production (P < 0.05). BCA treatment continued to maintain higher acetate concentrations than any other treatment during the SARA challenge periods and an acetate to propionate ratio that was similar to HF CON (P < 0.05).

It is widely accepted that rumen fermentation and the products produced are dependent on diet. Acetate to propionate ratio is generally lower in cereal grain fermentations as opposed to forages. Typically, these differences in dietary SCFA production are primarily attributed to the metabolic characteristics of fiber-degrading vs. starch-degrading bacteria. However, it has also been suggested that a reduction in pH alone can partially account for decreased acetate to propionate ratio by decreasing the viability of rumen methanogens and consequently impairing H_2_ reduction [56,61]. When a high carbohydrate diet is consumed, amylolytic bacteria proliferate and lactate is rapidly produced in the rumen. In SARA this lactate does not accumulate in the rumen environment because it is rapidly metabolized to propionate by resident lactate-fermenting bacteria [13]. Therefore, the negligible lactate concentrations and increased propionate concentrations detected in the current study with SARA challenge were expected. The observed increased concentrations of total SCFA and lower acetate to propionate ratio with SARA induction in the current study are consistent with fermentation of high-starch diets and the pathogenesis of SARA [2,3,6,56]. It is important to note, that although the total SCFA concentrations reported are comparable, the acetate to propionate ratio over the course of the current study was relatively low when compared to reported values in the literature [14,16,20]. This could be partly attributed to any of the aforementioned variables described above but is likely multifactorial and requires further investigation.

**Fig 1:**
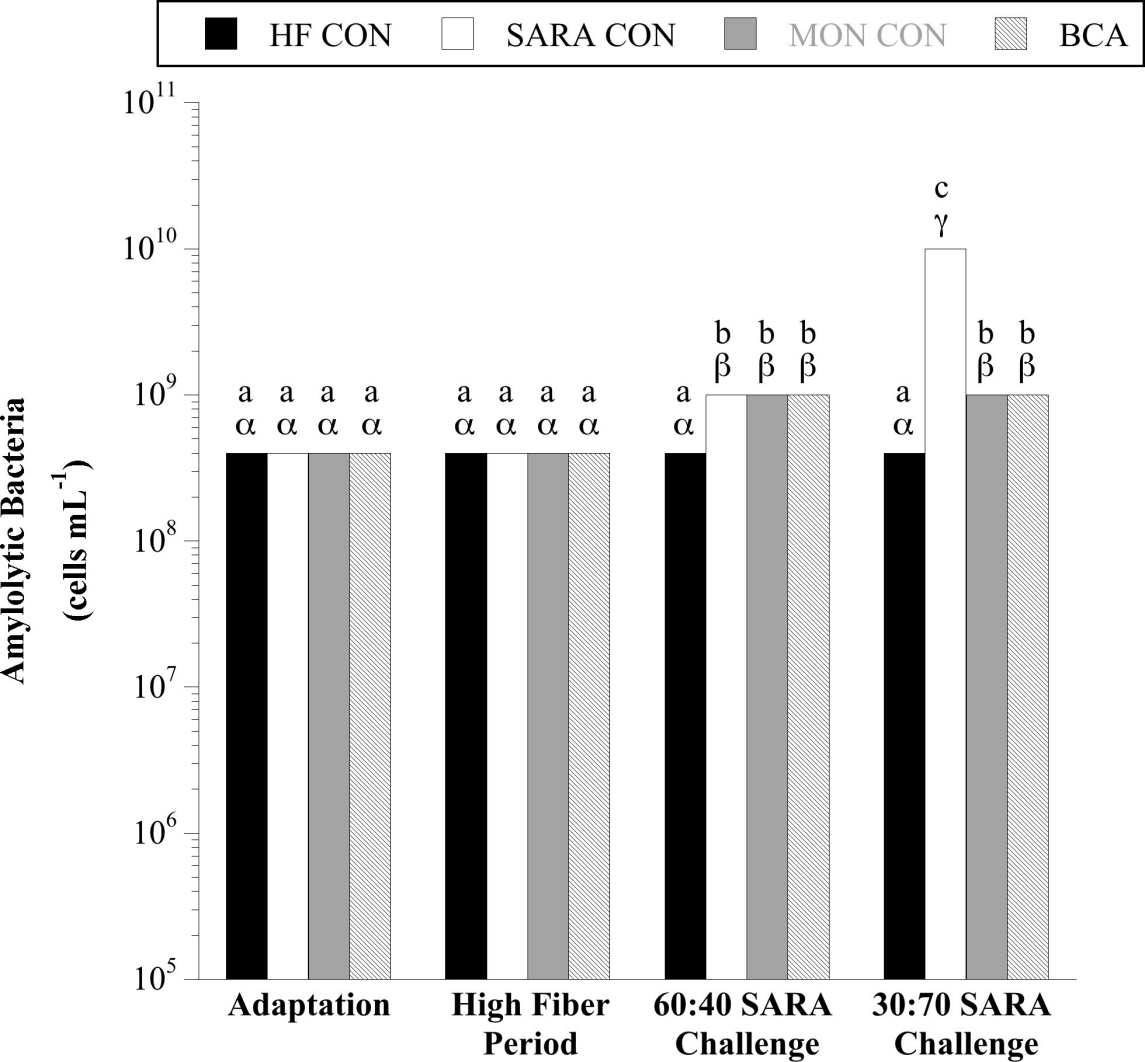
The effect of biochanin A on the viable number of amylolytic bacteria in rumen fluid (4 h post-feeding). Holstein steers (n = 12) were blocked by weight into 1 of 4 treatments: High fiber control (HF CON; basal diet control, corn silage + dried distillers’ grain, to meet protein requirements; n = 3), SARA control (SARA CON; n = 3), SARA + monensin treatment (MON CON; 200 mg d^-1^ monensin; n = 3), or SARA + biochanin A treatment (BCA; 6 g d^-1^ biochanin A; n = 3). Rumen fluid samples were taken at the end of the adaptation period (100% basal diet), high fiber period (100% basal diet + treatments), 60:40 SARA challenge period (60% basal diet + 40% cracked corn + treatments), and 30:70 SARA challenge period (30% basal diet + 70% cracked corn + treatments) for amylolytic bacterial enumeration. The enumerations were performed in anaerobic liquid media with soluble starch as the growth substrate. The tubes were incubated (39°C, 3 d), and the final dilution exhibiting bacterial growth (visual examination; OD_600_) was recorded as the viable number. Means lacking a common English letter are different within sample day (*P* < 0.05). Means lacking a common Greek letter are different over sample days within treatment (*P* < 0.05). Treatment: *P* = 0.0150, sample day: *P* = 0.0002, and treatment × sample day: *P* = 0.0680; Pooled SEM: Treatment = 0.0833, sample day = 0.1102, treatment × sample day = 0.2205 (log transformed).

When steers were consuming corn silage and dried distillers’ grain, 10^8^–10^9^ total viable amylolytic bacteria per mL rumen fluid were detected, regardless of treatment (Fig 1). There was a trend for a treatment × sample day interaction for the viable number of amylolytic bacteria (P = 0.07). When steers were challenged with 40% cracked corn, the total number of amylolytic bacteria increased 2.5-fold with no effect of MON or BCA treatment (P < 0.05). The viable number of amylolytics continued to increase 10-fold in the SARA control when the corn challenge was increased to 70% (P < 0.05). Both BCA and MON treatment were equally effective at preventing further proliferation of amylolytic bacteria when cracked corn was increased from 40% to 70% (P < 0.05).

**Fig 2:**
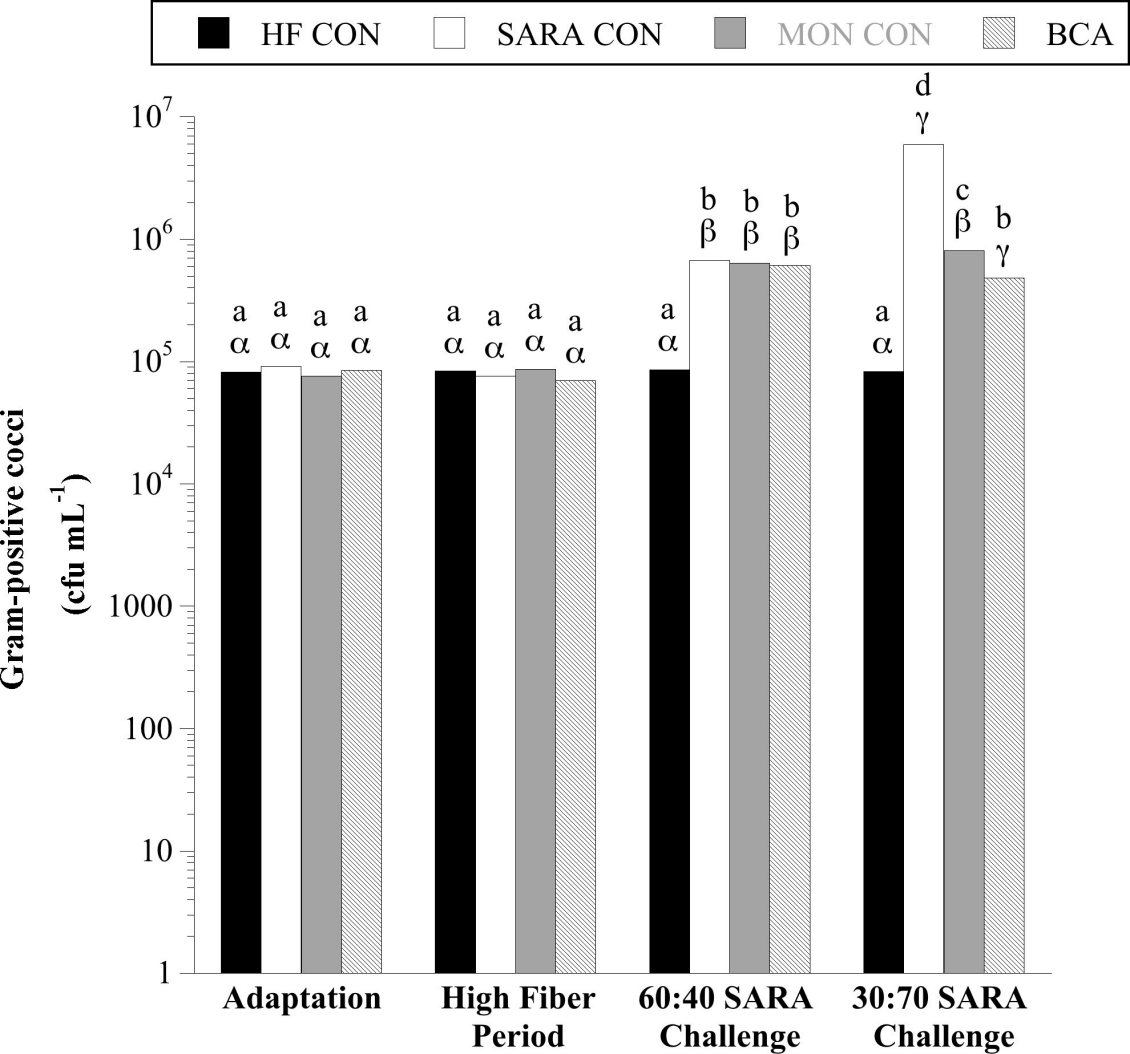
Effect of biochanin A on the viable number of Group D Gram-positive cocci (GPC; *Streptococcus bovis*, *Enterococcus* spp.) in rumen fluid (4 h post-feeding). Holstein steers (n = 12) were blocked by weight into 1 of 4 treatments: High fiber control (HF CON; basal diet control, corn silage + dried distillers’ grain, to meet protein requirements; n = 3), SARA control (SARA CON; n = 3), SARA + monensin treatment (MON CON; 200 mg d^-1^ monensin; n = 3), or SARA + biochanin A treatment (BCA; 6 g d^-1^ biochanin A; n = 3). Rumen fluid samples were taken at the end of the adaptation period (100% basal diet), high fiber period (100% basal diet + treatments), 60:40 SARA challenge period (60% basal diet + 40% cracked corn + treatments), and 30:70 SARA challenge period (30% basal diet + 70% cracked corn + treatments) for GPC bacterial enumeration. GPC were enumerated on bile esculin azide agar (BD). The plates were incubated aerobically (39°C, 3 d). Plates with 30 < x < 300 colonies were counted. Black colonies on bile esculin azide agar were counted as GPC. Means lacking a common English letter are different within sample day (*P* < 0.05). Means lacking a common Greek letter are different over sample days within treatment (*P* < 0.05). Treatment: *P* < 0.0001, sample day: *P* < 0.0001, and treatment × sample day: *P* < 0.0001; Pooled SEM: Treatment = 0.0156, sample day = 0.0192, treatment × sample day = 0.0385 (log transformed).

**Fig 3:**
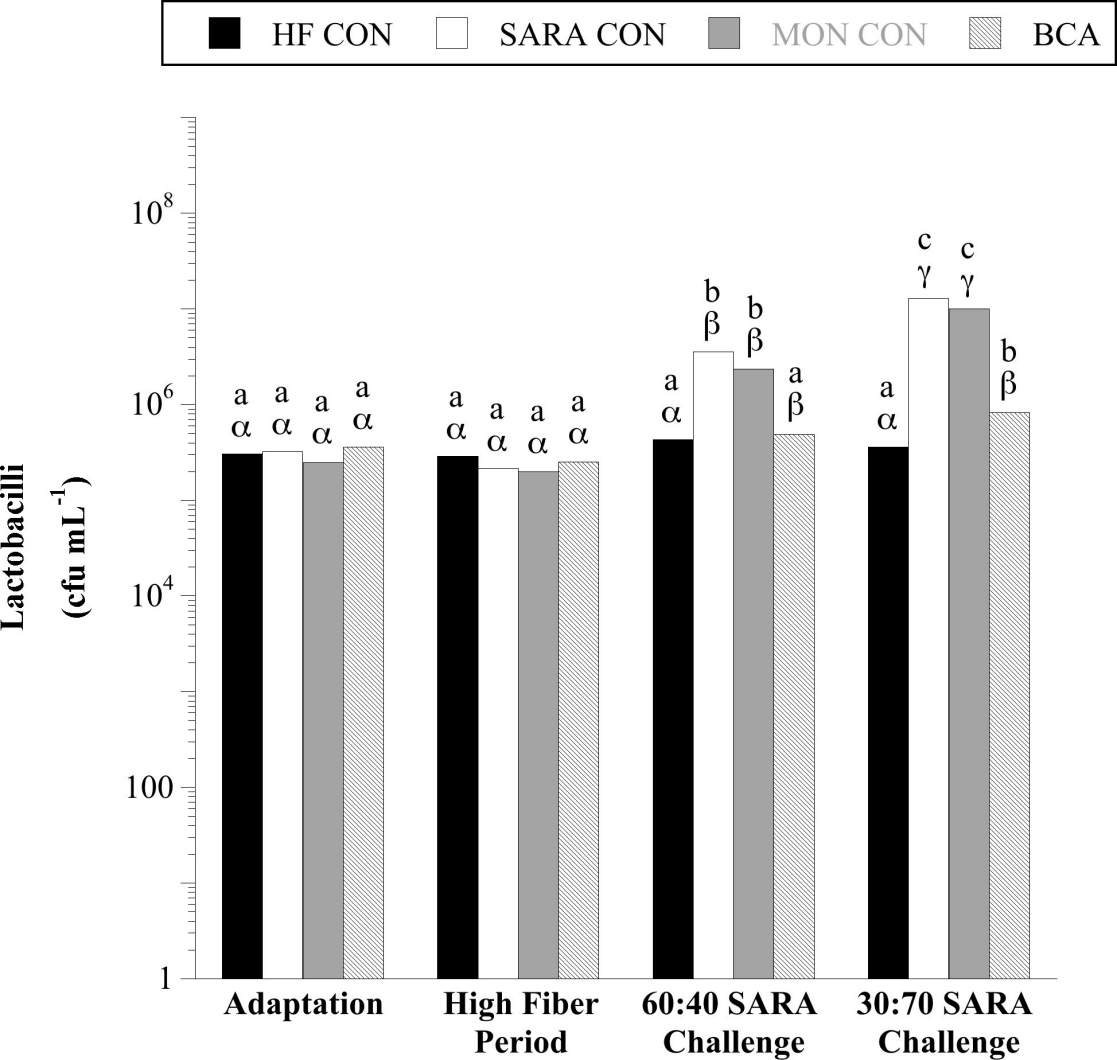
Effect of biochanin A on the viable number of lactobacilli in rumen fluid (4 h post-feeding). Holstein steers (n = 12) were blocked by weight into 1 of 4 treatments: High fiber control (HF CON; basal diet control, corn silage + dried distillers’ grain, to meet protein requirements; n = 3), SARA control (SARA CON; n = 3), SARA + monensin treatment (MON CON; 200 mg d^-1^ monensin; n = 3), or SARA + biochanin A treatment (BCA; 6 g d^-1^ biochanin A; n = 3). Rumen fluid samples were taken at the end of the adaptation period (100% basal diet), high fiber period (100% basal diet + treatments), 60:40 SARA challenge period (60% basal diet + 40% cracked corn + treatments), and 30:70 SARA challenge period (30% basal diet + 70% cracked corn + treatments) for lactobacilli bacterial enumeration. The enumerations were performed on Rogosa SL agar (BD). The plates were incubated aerobically (39°C, 3 d). Plates with 30 < x < 300 colonies were counted. All colonies on Rogosa SL agar were counted as lactobacilli. Means lacking a common English letter are different within sample day (*P* < 0.05). Means lacking a common Greek letter are different over sample days within treatment (*P* < 0.05). Treatment: *P* < 0.0001, sample day: *P* < 0.0001, and treatment × sample day: *P* < 0.0001; Pooled SEM: Treatment = 0.0352, sample day = 0.0398, treatment × sample day = 0.0796 (log transformed).

Results similar to total amylolytics were also observed for two broad phylogenetic groups within the amylolytic guild, Gram-positive cocci (GPC; Fig 2) and lactobacilli (Fig 3). When steers were consuming the basal diet, on average 8.34 × 10^4^ and 3.3 × 10^5^ CFU per mL total GPC and lactobacilli were enumerated, respectively. There was a treatment × sample day interaction for both GPC and lactobacilli (*P* < 0.0001, in both cases). When steers were challenged with a 40% cracked corn diet, GPC and lactobacilli proliferated and continued to increase when the diet was stepped up to 70% cracked corn (*P* < 0.05). There was no effect of MON treatment on GPC or lactobacilli when steers consumed the basal diet or the 60:40 SARA challenge diet (*P* > 0.05). During the 30:70 SARA challenge, MON treatment partially inhibited the GPC proliferation compared to SARA CON steers (*P* < 0.05) but had no effect on the viable number of lactobacilli (*P* > 0.05). Similarly, there was no effect of BCA during the HF period for either amylolytic population or on the viable number of GPC during the 60:40 SARA challenge period (*P* > 0.05). Biochanin A treatment partially mitigated the proliferation of lactobacilli in both SARA challenge periods and GPC in the 30:70 SARA challenge period (P < 0.05).

### Cellulolytic bacteria and *In situ* DM disappearance

**Fig 4:**
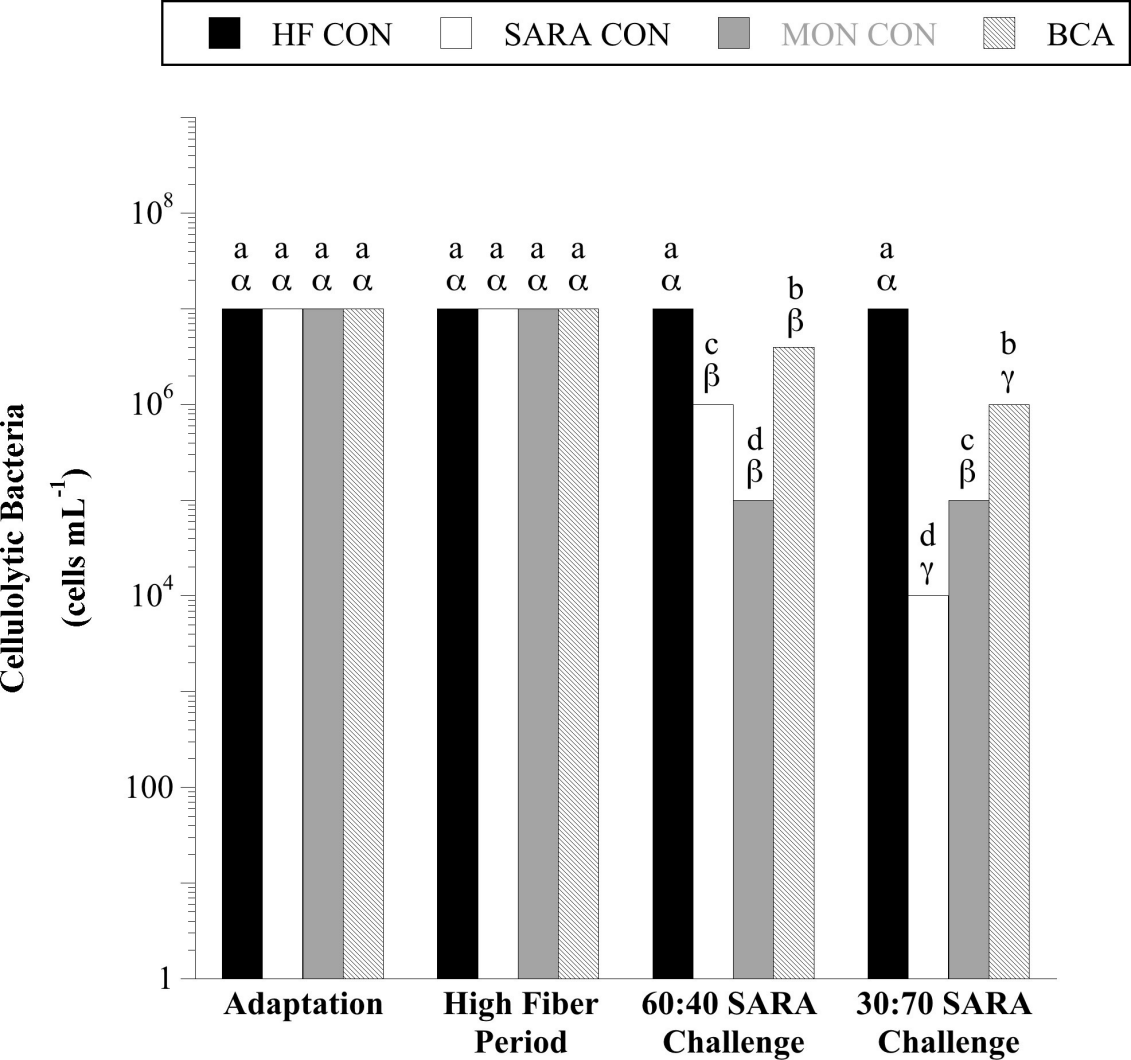
Effect of biochanin A on the viable number of total cellulolytic bacteria in rumen fluid (4 h post-feeding). Holstein steers (n = 12) were blocked by weight into 1 of 4 treatments: High fiber control (HF CON; basal diet control, corn silage + dried distillers’ grain, to meet protein requirements; n = 3), SARA control (SARA CON; n = 3), SARA + monensin treatment (MON CON; 200 mg d^-1^ monensin; n = 3), or SARA + biochanin A treatment (BCA; 6 g d^-1^ biochanin A; n = 3). Rumen fluid samples were taken at the end of the adaptation period (100% basal diet), high fiber period (100% basal diet + treatments), 60:40 SARA challenge period (60% basal diet + 40% cracked corn + treatments), and 30:70 SARA challenge period (30% basal diet + 70% cracked corn + treatments) for total cellulolytic bacterial enumeration. The enumerations were performed in anaerobic liquid media with cellulose (Whatman #1 filter paper strips) as the growth substrate. The tubes were incubated (39°C, 10 d), and the final dilution exhibiting dissolution of cellulose (visual examination) was recorded as the viable number. Means lacking a common English letter are different within sample day (*P* < 0.05). Means lacking a common Greek letter are different over sample days within treatment (*P* < 0.05). Treatment: *P* < 0.0001, sample day: *P* < 0.0001, and treatment × sample day: *P* < 0.0001; Pooled SEM: Treatment = 0.0417, sample day = 0.0417, treatment × sample day = 0.0833 (log transformed).

When steers consumed corn silage with supplemental dried distillers’ grains (to meet protein requirements), 10^7^ viable cells per mL total cellulolytic bacteria were detected, regardless of MON or BCA treatment (Fig 4). A treatment × sample day interaction was observed for the viable number of total cellulolytic bacteria (*P* < 0.0001). Cellulolytic bacteria were inhibited 10-fold (10^6^ viable cells per mL) and an additional 100-fold (10^4^) during the 60:40 and 30:70 SARA challenge periods, respectively (*P* < 0.05). When steers were treated with MON, an even greater inhibition of total cellulolytic bacteria was observed (100-fold, 10^5^ viable cells per mL) during the 60:40 challenge (*P* < 0.05), but no further inhibition occurred in the 30:70 SARA challenge period (*P* > 0.05). In contrast BCA treatment was able to partially mitigate the decline in viable cellulolytic bacteria associated with both levels of SARA challenge (*P* < 0.05).

The inhibitor 2-deoxy-D-glucose (2-DG) is a non-metabolizable glucose analog that inhibits phosphorylation of glucose by hexokinase, the first step of glycolysis. Historically, 2-DG has been used to study carbohydrate metabolism and transport by cellulolytic microorganisms [62,63], but has also been used as a selective agent for the isolation of glucose de-regulated, highly cellulolytic (high cellulase producing), microorganisms [64–67]. Therefore, 2-DG was utilized in the current study as a media addition to enumerate a subset of strictly cellulolytic bacteria from the rumen. These cellulolytic organisms that do not transport monomeric glucose are a subset of the cellulolytic guild in the same way that lactobacilli and GPC are subsets of the amylolytic guild.

**Fig 5:**
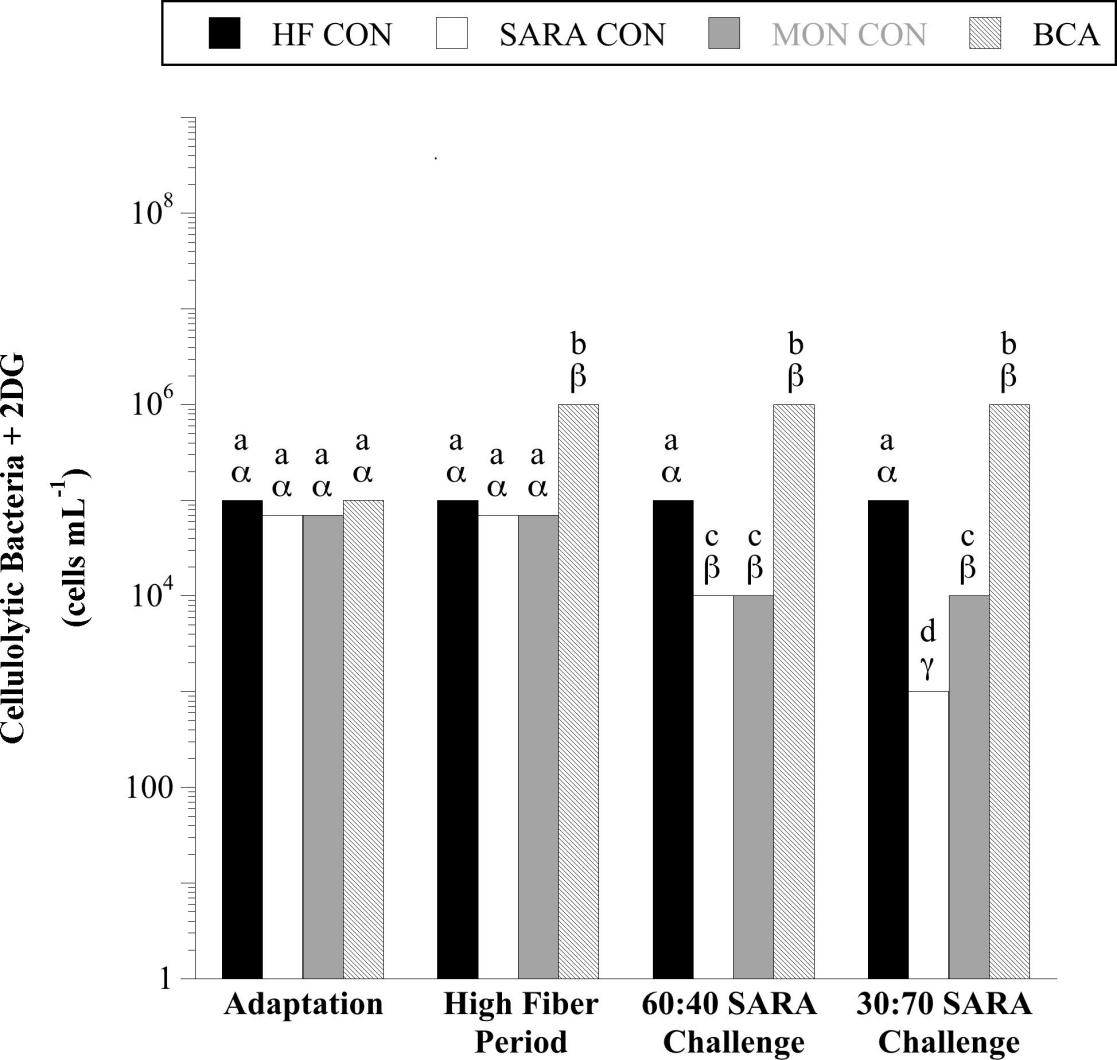
Effect of biochanin A on the viable number of 2-deoxy-D-glucose (2-DG) resistant cellulolytic bacteria in rumen fluid (4 h post-feeding). Holstein steers (n = 12) were blocked by weight into 1 of 4 treatments: High fiber control (HF CON; basal diet control, corn silage + dried distillers’ grain, to meet protein requirements; n = 3), SARA control (SARA CON; n = 3), SARA + monensin treatment (MON CON; 200 mg d^-1^ monensin; n = 3), or SARA + biochanin A treatment (BCA; 6 g d^-1^ biochanin A; n = 3). Rumen fluid samples were taken at the end of the adaptation period (100% basal diet), high fiber period (100% basal diet + treatments), 60:40 SARA challenge period (60% basal diet + 40% cracked corn + treatments), and 30:70 SARA challenge period (30% basal diet + 70% cracked corn + treatments) for 2-DG resistant cellulolytic bacterial enumeration. The enumerations were performed in anaerobic liquid media with cellulose (Whatman #1 filter paper strips) as the growth substrate with added 2-DG (6 mmol L^-1^). The tubes were incubated (39°C, 10 d), and the final dilution exhibiting dissolution of cellulose (visual examination) was recorded as the viable number. Means lacking a common English letter are different within sample day (*P* < 0.05). Means lacking a common Greek letter are different over sample days within treatment (*P* < 0.05). Treatment: *P* < 0.0001, sample day: *P* < 0.0001, and treatment × sample day: *P* < 0.0001; Pooled SEM: Treatment = 0.1273, sample day = 0.0867, treatment × sample day = 0.1735 (log transformed).

Steers consuming the basal diet on average had 9.1 × 10^4^ 2-DG resistant cellulolytic bacteria, approximately 1% of the total cellulolytic bacteria (Fig 5). There was a treatment × sample day interaction detected for 2-DG resistant cellulolytic bacteria (*P* < 0.0001). Treatment with BCA during the HF period increased the viable number of 2-DG resistant cellulolytic bacteria >10-fold (10^6^ viable cells per mL; *P* < 0.05). Similarly, to what was observed in total cellulolytic bacteria enumerations, 2-DG resistant cellulolytic bacteria were inhibited by 60:40 SARA challenge (10^4^), with even greater inhibition observed with 30:70 SARA challenge (10^3^; *P* < 0.05). Treatment with MON had no effect on 2-DG resistant cellulolytics during the 60:40 SARA challenge period (same as SARA CON), but partially mitigated their inhibition in 30:70 SARA challenge (+10-fold compared to SARA CON; P < 0.05). This latter result suggests that the 2-DG resistant species might be more sensitive to acidic pH or fermentation acids than to the inhibitor, monensin. Treatment with BCA maintained 10^6^ total 2-DG resistant cellulolytic bacteria despite SARA challenge (*P* < 0.05). These results indicate that not only does BCA treatment mitigate declines in viable cellulolytic bacteria during SARA, it also promotes a sub-population of cellulolytic 2-DG resistant bacteria, even when steers were consuming the basal diet with no added cracked corn.

Please note that results obtained through broth dilution and growth of fibrolytic microorganism must be interpreted critically. Many cellulolytic and more generally fibrolytic bacteria can adhere to the fiber. In the method used, only those fiber particles small enough to pass through 3 layers of cheesecloth and an 18-gauge needle are added to the media. Thus, fiber-adherent microorganisms will be underrepresented. Furthermore, any growth medium is limited by the nutritional requirements of the microorganisms; i.e., only those which nutritional requirements are met by the medium will grow. Nevertheless, culture-based enumeration has a distinct advantage over the culture independent molecular methods commonly used today. The relationships inferred by alignment of 16S ribosomal subunit sequences are phylogenetic. They do not necessarily indicate the physiology or ecological roles of the microorganisms. However, when an organism grows on and dissolves cellulose, then it is clear that the organism is cellulolytic, regardless of its phylogenetic identity.

**Fig 6:**
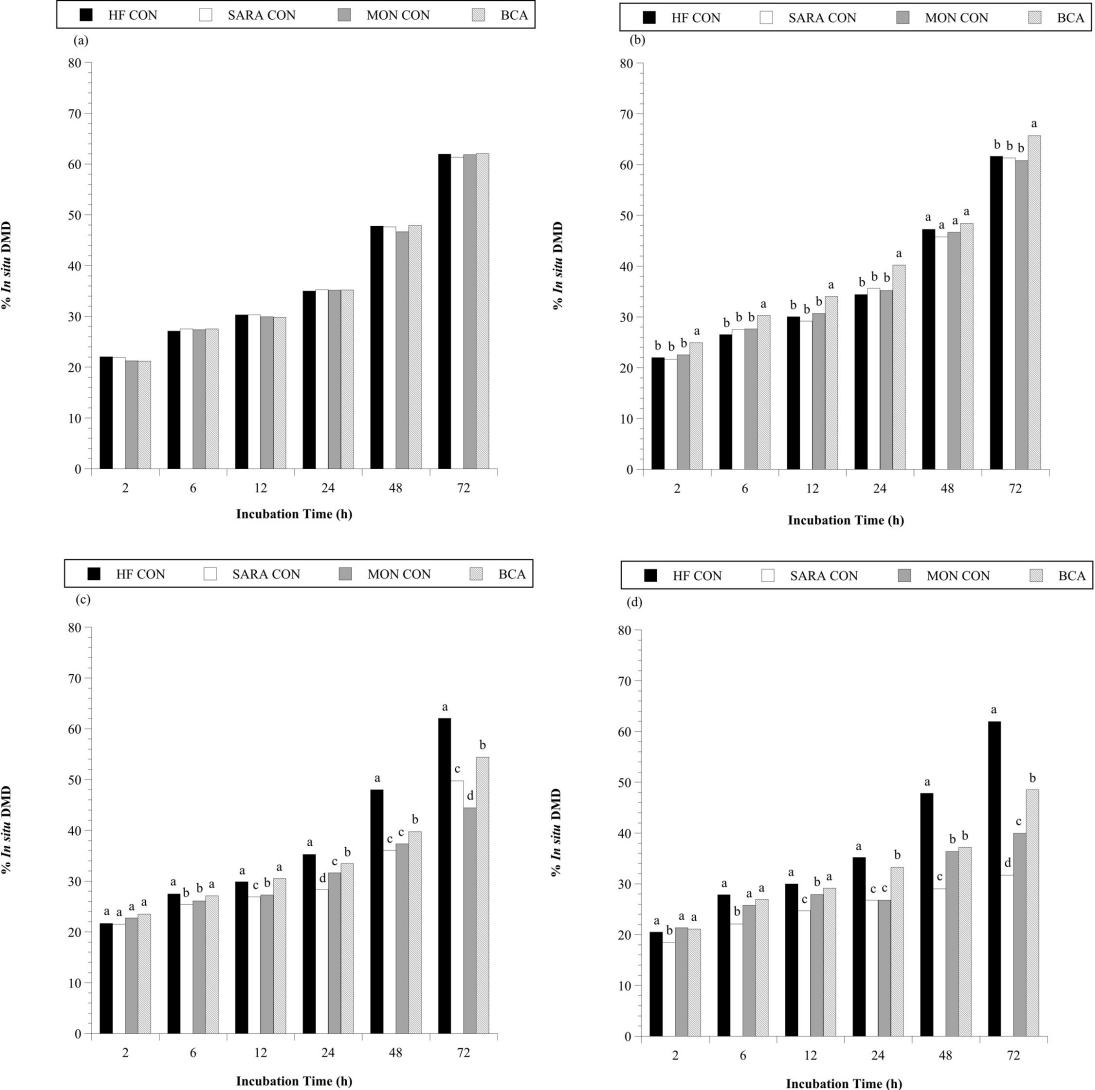
Effect of biochanin A addition on the *in situ* DM disappearance (% *in situ* DMD) of ground high quality grass hay in the steer rumen. Holstein steers (n = 12) were blocked by weight into 1 of 4 treatments: High fiber control (HF CON; basal diet control, corn silage + dried distillers’ grain, to meet protein requirements; n = 3), SARA control (SARA CON; n = 3), SARA + monensin treatment (MON CON; 200 mg d^-1^ monensin; n = 3), or SARA + biochanin A treatment (BCA; 6 g d^-1^ biochanin A; n = 3). *In situ* Dacron bags were added at 0, 24, 48, 60, 66, and 70 h of incubation into a weighted mesh bag in the rumen and removed together at 72 h on the last day of the (a) adaptation period (100% basal diet) (b) high fiber period (100% basal diet + treatments), (c) 60:40 SARA challenge period (60% basal diet + 40% cracked corn + treatments, as fed basis), and the (d) 30:70 SARA challenge period (30% basal diet + 70% cracked corn + treatments, as fed basis). Means lacking a common English letter are different within incubation time (*P* < 0.05); Treatment: *P* < 0.0001; Pooled SEM: Treatment = 0.8882.

**Fig 7:**
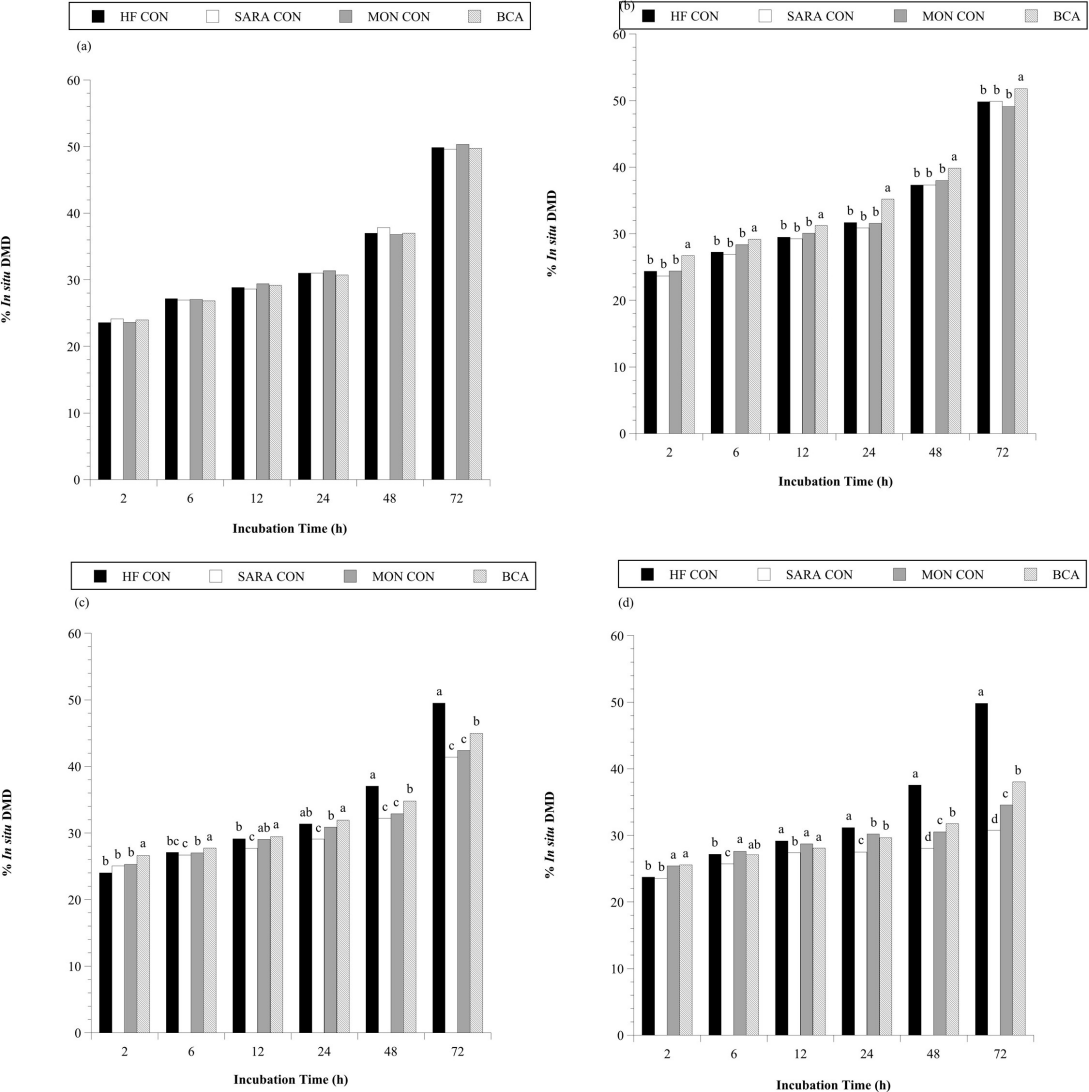
Effect of biochanin A addition on the *in situ* DM disappearance (% *in situ* DMD) of ground low quality grass hay in the steer rumen. Holstein steers (n = 12) were blocked by weight into 1 of 4 treatments: High fiber control (HF CON; basal diet control, corn silage + dried distillers’ grain, to meet protein requirements; n = 3), SARA control (SARA CON; n = 3), SARA + monensin treatment (MON CON; 200 mg d^-1^ monensin; n = 3), or SARA + biochanin A treatment (BCA; 6 g d^-1^ biochanin A; n = 3). *In situ* Dacron bags were added at 0, 24, 48, 60, 66, and 70 h of incubation into a weighted mesh bag in the rumen and removed together at 72 h on the last day of the (a) adaptation period (100% basal diet) (b) high fiber period (100% basal diet + treatments), (c) 60:40 SARA challenge period (60% basal diet + 40% cracked corn + treatments, as fed basis), and the (d) 30:70 SARA challenge period (30% basal diet + 70% cracked corn + treatments, as fed basis). Means lacking a common English letter are different within incubation time (*P* < 0.05); Treatment: *P* < 0.0001; Pooled SEM: Treatment = 0.4265.

In addition to increasing the viable number of cellulolytic bacteria, BCA treatment also increased *in situ* disappearance of high (Fig 6A–6D) and low-quality hay (Fig 7A–7D). An effect of treatment was detected for the *in situ* dry matter disappearance of both the high- and low-quality hay substrates (*P* < 0.0001, in both cases). When steers consumed the basal diet only, the extent of *in situ* degradation after 72 h of incubation were 62% and 50% for high- (Fig 6A) and low- (Fig 7A) quality hay, respectively. Treatment with BCA during the HF period increased *in situ* DMD of high-quality hay after 2 (+3%), 6 (+3%), 12 (+4%), 24 (+5%) and 72 h (+4.5%) of rumen incubation (Fig 6B; *P* < 0.05). BCA treatment also increased *in situ* DMD of the low-quality hay substrate 2–4% at all time points during the HF period (Fig 7B; *P* < 0.05). In contrast, MON treatment had no impact on *in situ* DMD of hay when steers consumed the basal diet (*P* > 0.05). The effect of BCA on *in situ* DMD observed may be partially attributed to the observation that more, and/or compositionally different, cellulolytic microbiota were present in the rumen of steers given BCA.

Addition of cracked corn to the diet decreased *in situ* DMD of high-quality hay by 12 and 30% compared to the HF CON treatment during the 60:40 (Fig 6C) and 30:70 (Fig 6D) SARA challenge, respectively (72 h of rumen incubation). Similar, but let dramatic, results were observed with the low-quality hay substrate after 72 h of rumen incubation (8% less, 60:40 SARA challenge, Fig 7C; 19% less, 30:70 SARA challenge, Fig 7D; *P* < 0.05, in all cases). Treatment with MON had variable results on *in situ* DMD during the 60:40 SARA challenge period. At 6, 12 and 24 h of rumen incubation (excluding 6 h with high quality hay) MON treatment partially mitigated DMD decline associated with the SARA challenge (+0.3–3%, *P* < 0.05, in all cases). In contrast, at 72 h of incubation MON treated steers had 5% lower *in situ* DMD of high-quality hay than SARA CON steers (*P* < 0.05). A hypothesis to explain this latter result is that many generalist species, such as the numerically dominant, Gram-negative *Prevotella* species, can degrade NDF components [68]. The Gram-negative cell envelope makes these species more resistant to monensin [26]. However, to degrade more recalcitrant fibers left at 72 h would require different species, including the Gram-positive and monensin-sensitive ruminococci. During the 30:70 SARA challenge period, MON treatment partially mitigated *in situ* DMD decline at all time points for both hay substrates (excluding 24 h with high quality hay; +1.3–8.3%; *P* < 0.05, in all cases). Biochanin A treatment partially mitigated *in situ* DMD decline associated with SARA challenge at all time points regardless of challenge period or hay substrate (excluding 2 h with high quality hay during the 60:40 SARA challenge period; *P* < 0.05, in all cases). After 72 h of rumen incubation, *in situ* DMD of high-quality hay in BCA treated steers was 4.6% and 16.8% higher than SARA CON steers during the 60:40 and 30:70 SARA challenge periods, respectively (P < 0.05, in all cases). Similar improvements of *in situ* DMD were also observed with the low-quality hay substrate (after 72 h of incubation +3.5% and +7.3% during the 60:40 and 30:70 SARA challenge periods, respectively; *P* < 0.05, in all cases). Additionally, after 72 h of incubation, BCA treatment was consistently more effective than MON at mitigating *in situ* DMD decline with SARA challenge (*P* < 0.05. in all cases).

Cellulose is the most abundant carbohydrate in the world [69]. Furthermore, enzymes required for cellulose degradation are strictly microbial. Therefore, cellulolytic microbes are arguably the most important metabolic guild in the rumen. Cellulolytic bacteria in the rumen are highly sensitive to changes in environmental pH [8–10]. As expected, in the current study, both SARA challenge periods elicited rumen pH decline, cellulolytic bacteria inhibition, and reduced high- and low-quality hay *in situ* DMD in comparison to HF CON.

As mentioned previously, a disadvantage of commonly utilized feed antibiotics (e.g., ionophores like monensin) is that they have a broad spectrum of activity which include rumen cellulolytic bacteria. Therefore, their efficacy and benefits to both the health and performance of the animal are often considered to be diet dependent. For example, ionophore antibiotics do not consistently increase and oftentimes can actually decrease growth performance in cattle consuming high-fiber diets like those in pasture-based systems [70]. In contrast, research has demonstrated that when animals consume high-starch diets, like those in finishing systems, ionophore antibiotics consistently have both health and performance benefits [24,26,71–75]. When steers consumed the high-fiber basal diet in the current study, there was no effect of MON treatment on the viable number of cellulolytic bacteria or the digestibility of either hay substrate. However, during the 60:40 SARA challenge period, steers supplemented with MON had fewer viable cellulolytic bacteria (100-fold) detected and lower *in situ* hay digestibility (-5%, HQ hay, 72 h) than control steers that consumed the same diet, but with no supplement. Therefore, consistent with previous research, MON treatment in the current study was not beneficial when steers consumed diets that were higher in fiber. However, when steers were challenged with a 70% cracked corn diet in the current study (similar to feedlot rations), MON treatment partially mitigated cellulolytic inhibition and improved high- and low-quality hay digestibility. Monensin, like other, more specific protonophores, is more potent at lower pH values [76]. The inhibition of sensitive microbial species decreases the total extentof fermentation and the higher pH and lower concentration of fermentation acids might spare some acid-sensitive, monensin-insensitive fibrolytic species.

Across all experimental conditions, BCA treatment provided greater benefits to the stability and presumably improved fermentative efficiency of the rumen cellulolytic bacterial community when compared to MON. During the HF period, BCA treatment selected for a metabolically different cellulolytic bacterial guild (+2-DG resistant) and demonstrated increased utilization of both the high- and low-quality hay substrates *in situ* (+2–5% DMD). *In situ* DMD of the fiber component specifically was not evaluated in the current study. However, these results are consistent with previous work from our laboratory that demonstrated that BCA treatment can improve cellulolytic fermentative efficiency presumably by ecological manipulation of the cellulolytic guild in an *ex vivo* rumen model [42]. Additionally, field experiments conducted by our research group have demonstrated increased *ex vivo* fiber degradation (+10–25% DMD) and average daily gains (+0.1–0.2 kg d^-1^) when BCA was supplemented in a purified form or via red clover hay to steers grazing mixed grass pastures in comparison to protein-matched controls [41,43]. Therefore, the improvement of *in situ* DMD of hay with BCA treatment observed in the current study could be result of improved fiber digestibility. Future research is needed to better elucidate the relationships between rumen microbial community structure and function with BCA treatment.

During both SARA challenge periods BCA treatment was most effective at partially mitigating cellulolytic bacteria inhibition and decreased *in situ* DMD of hay observed in the SARA CON steers. In addition to ecologically altering and apparently optimizing the cellulolytic guild, it is likely that the improvement in *in situ* DMD of hay with BCA treatment may be attributed to the less acidic pH during the SARA challenge periods.

### Protein-, peptide- and amino acid-utilizing bacteria

**Fig 8:**
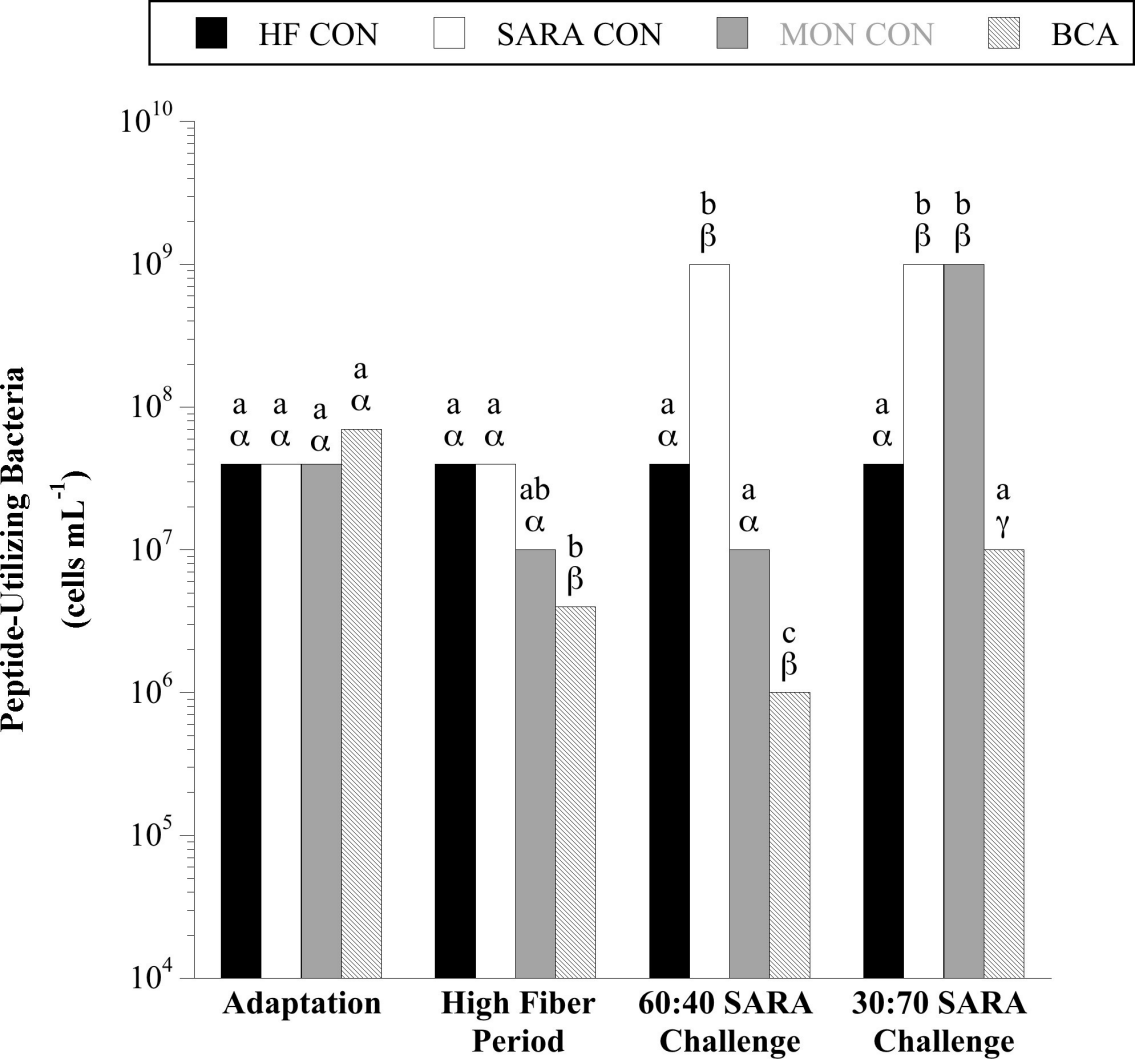
Effect of biochanin A on the viable number of peptide-utilizing bacteria in rumen fluid (4 h post-feeding). Holstein steers (n = 12) were blocked by weight into 1 of 4 treatments: High fiber control (HF CON; basal diet control, corn silage + dried distillers’ grain, to meet protein requirements; n = 3), SARA control (SARA CON; n = 3), SARA + monensin treatment (MON CON; 200 mg d^-1^ monensin; n = 3), or SARA + biochanin A treatment (BCA; 6 g d^-1^ biochanin A; n = 3). Rumen fluid samples were taken at the end of the adaptation period (100% basal diet), high fiber period (100% basal diet + treatments), 60:40 SARA challenge period (60% basal diet + 40% cracked corn + treatments), and 30:70 SARA challenge period (30% basal diet + 70% cracked corn + treatments) for peptide-utilizing bacteria enumeration. The enumerations were performed in anaerobic liquid media with Trypticase (BD) as the growth substrate. The tubes were incubated (39°C, 3 d), and the final dilution exhibiting growth (visual examination, OD_600_) was recorded as the viable number. Means lacking a common English letter are different within sample day (*P* < 0.05). Means lacking a common Greek letter are different over sample days within treatment (*P* < 0.05). Treatment: *P* = 0.0242, sample day: *P* < 0.0001, and treatment × sample day: *P* < 0.0001; Pooled SEM: Treatment = 0.2269, sample day = 0.1345, treatment × sample day = 0.2690 (log transformed).

**Fig 9:**
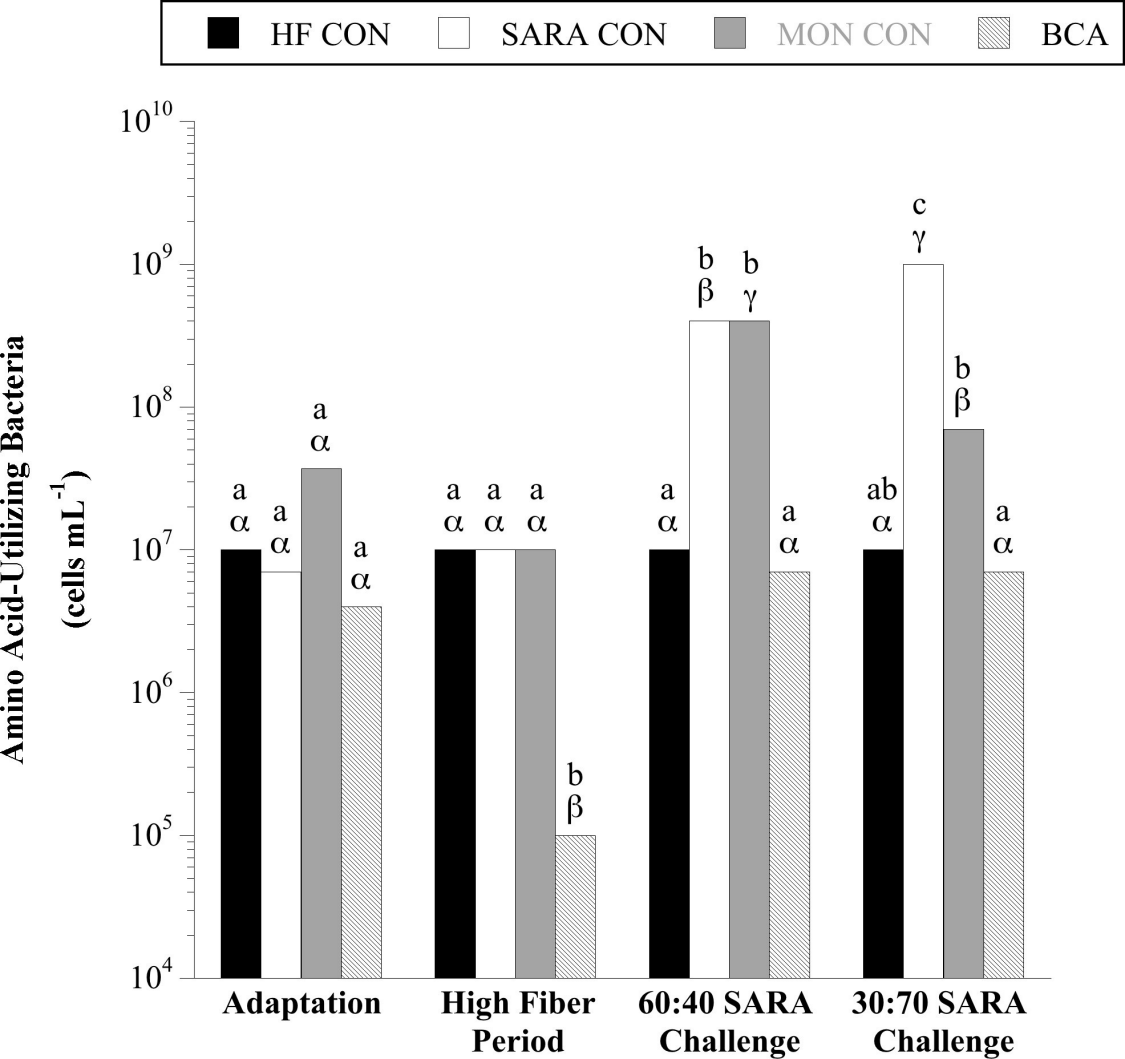
Effect of biochanin A on the viable number of amino acid-utilizing bacteria in rumen fluid (4 h post-feeding). Holstein steers (n = 12) were blocked by weight into 1 of 4 treatments: High fiber control (HF CON; basal diet control, corn silage + dried distillers’ grain, to meet protein requirements; n = 3), SARA control (SARA CON; n = 3), SARA + monensin treatment (MON CON; 200 mg d^-1^ monensin; n = 3), or SARA + biochanin A treatment (BCA; 6 g d^-1^ biochanin A; n = 3). Rumen fluid samples were taken at the end of the adaptation period (100% basal diet), high fiber period (100% basal diet + treatments), 60:40 SARA challenge period (60% basal diet + 40% cracked corn + treatments), and 30:70 SARA challenge period (30% basal diet + 70% cracked corn + treatments) for amino acid-utilizing bacteria enumeration. The enumerations were performed in anaerobic liquid media with Casamino acids (BD) as the growth substrate. The tubes were incubated (39°C, 3 d), and the final dilution exhibiting growth (visual examination, OD_600_) was recorded as the viable number. Means lacking a common English letter are different within sample day (*P* < 0.05). Means lacking a common Greek letter are different over sample days within treatment (*P* < 0.05). Treatment: *P* = 0.0026, sample day: *P* < 0.0001, and treatment × sample day: *P* = 0.0005; Pooled SEM: Treatment = 0.1718, sample day = 0.1382, treatment × sample day = 0.2764 (log transformed).

Over the course of the experiment, 102–10^3^ gelatin-hydrolyzing bacteria per mL rumen fluid were detected regardless of diet or treatment (*P* = 0.17; S1 Fig). When steers consumed the basal diet of corn silage and dried distillers’ grains 10^7^–10^8^, and 10^6^−10^8^ total peptide- (Fig 8) and amino acid-utilizing (Fig 9) bacteria were detected per mL rumen fluid, respectively. A treatment × sample day interaction was observed for the total viable number of peptide- (*P* < 0.0001) and amino acid-utilizing (*P* < 0.01) bacteria. During the HF period, BCA treatment decreased peptide- and amino acid- utilizing bacteria 10-fold and 100-fold, respectively (*P* < 0.05). In contrast, MON treatment had no effect during the HF period (*P* > 0.05). The viable numbers of peptide- and amino acid-utilizing bacteria increased 10–100-fold with SARA challenge (*P* < 0.05). Monensin treatment effectively inhibited the proliferation of peptide-utilizing bacteria and amino acid-utilizing bacteria during the 60:40 and 30:70 SARA challenge periods, respectively (*P* < 0.05); but remained similar to SARA CON steers in all other cases (*P* > 0.05). In contrast, BCA treatment inhibited the proliferation of peptide- and amino acid-utilizing bacteria in both SARA challenge periods to be lower (*P* < 0.05) or equal (*P* > 0.05) in viable number to the HF CON treatment.

In a manner that is analogous to cellulose degradation, proteolytic microflora in the rumen produce extracellular enzymes that convert consumed dietary proteins into peptides and amino acids [77]. The free peptides and amino acids can then by utilized to make microbial protein or deaminated to ammonia, which can also be assimilated to meet microbial requirements [78]. However, when the rate of ammonia production exceeds the rate of assimilation, ammonia is absorbed into the blood and lost as urea in the urine. Many rumen bacteria are proteolytic, but excess ammonia production is primarily attributed to resident APB [79]. The APB guild includes both peptide- and amino acid-utilizing bacteria, but all species are characterized by having rapid rates of ammonia production and a limited or no ability to utilize sugars. Amino acid/peptide-fermenting bacteria bacteria have been isolated from the rumen and identified in cattle [49,80] and other ruminant species [81,82].

Unlike the enzymes required for fiber degradation, ruminants produce mammalian enzymes necessary for catabolizing and utilizing protein; therefore, protein degradationby rumen microflora is considered to be a source of nutritional inefficiency. Decades of research has demonstrated that MON and other ionophores can have an “amino acid-sparing” effects by inhibiting APB in the rumen [28,80], but these effects can be animal and diet dependent [83,84]. In the current experiment MON treatment inhibited peptide- and amino acid-utilizing bacteria in some cases with SARA challenge, but not consistently. In contrast, BCA treatment reduced both peptide- and amino acid-utilizing bacteria on all sample days. Despite differences observed with peptide- and amino acid-utilizing bacteria, gelatin-hydrolyzing bacteria were unaffected by treatment. The gelatin media liquification method is commonly used in microbiology. However, it captures only gelatin-hydrolyzing species and not necessarily those that can hydrolyze other proteins such as the zein, gluten and rubisco abundant in the study diets. Also, note that the enumeration methods utilized in the current experiment do not account for other microorganisms, such as ciliates, that might be contributing to nutrient fermentation in the rumen including protein. Nevertheless, the results of the current study support the long-standing hypothesis that proteolysis and amino acid-deamination are distinct metabolic steps and are carried out by distinct microbial groups in the rumen [77]. Overall, these results are consistent with 10-years of experiments that have demonstrated that the spectrum of BCA antimicrobial activity includes rumen APB [38,40–43,85]. The current study is the first to show that amino acid- and peptide-fermenting bacteria are inhibited by the isoflavone in a high-starch diet.

## Conclusions

There is no indication that isoflavones, like BCA, have ionophore-like mechanisms of action [38]. Ionophores act by depolarizing the cell membrane [26]. Many phytochemical phenolic compounds do in fact share an ionophore-like mechanism of action [86]. Instead, BCA potentiates the antimicrobial activity of bacteriocins and possibly other rumen-endogenous antimicrobials [40]. This mode of action is consistent with results that show isoflavones can interfere with multidrug efflux pumps in some bacterial species [87,88].

The difference in mechanism of antimicrobial action between BCA and ionophores leads to a different spectrum of activity that would be advantageous in more than one production setting. The current study indicates that BCA could ameliorate pH and microbial changes in the rumen associated with high-starch diet consumption. It is also beneficial that amino acid-catabolizing bacteria, such as APB, are inhibited. It has long been recognized that rumen ammonia production can be reduced to increase bypass amino-nitrogen without limiting ammonia or branched chain VFA to the fibrolytic bacteria that require them [89]. BCA has the additional benefit of more limited inhibition of the cellulolytic bacteria [42]. In multiple *in vitro* and *in vivo* experiments, BCA has promoted net fiber utilization [42,43; current results]. These results stand in contrast to commonly used ionophores, which can negatively impact animal growth on high-fiber, forage-based diets [69]. It is important to note that monensin has veterinary use beyond antimicrobial growth promotion (i.e., it functions as a coccidostat), which can be important in many circumstances. The promotion of fiber degradation by a rumen microbiota exposed to BCA makes this compound advantageous in both high-starch and high-fiber diets.

The current experiment was conducted with purified BCA, which is not currently economical. However, supplemented grazing experiments indicated that red clover hay contained enough BCA to decrease APB, promote *ex vivo* DMD and ADG in steers [43]. Isoflavones, including BCA, can be found in many legume species (red clover, white clover, alfalfa, etc.). However, both isoflavone concentrations and profiles can vary between species. For example, although both red clover and white clover have similar isoflavone profiles (predominantly biochanin A and formononetin), red clover contains 200× more total isoflavones than white clover [90]. Research has demonstrated that red clover contains the highest concentrations of biochanin A, but isoflavone concentrations can vary depending on maturity and cultivar [85,91]. A survey of biochanin A concentrations in 10 cultivars of red clover demonstrated that concentrations can range from 4,081 to 5,332 μg g^−1^ DM [91].

The effects of BCA as a highly selective antimicrobial in both high-starch and high-fiber diets indicate its potential utility in both backgrounding and finishing systems. Steers were only subjected to a short corn step-up challenge in the current experiment. Although, these results clearly demonstrate advantages of BCA supplementation in cattle transitioning from high-fiber to high-starch diets, further research is needed to evaluate the efficacy of BCA supplementation over a longer period (>100 d) more relative to standard finishing systems. These results also highlight the potential roles of legumes and legume-based supplements in hybrid background/finishing systems. These systems might include pasture finishing as well as feedlot housing that incorporates the advantages of forage.

## Supporting information

S1 Fig Effect of biochanin A on the viable number of gelatin-hydrolyzing bacteria in rumen fluid (4 h post-feeding). Holstein steers (n = 12) were blocked by weight into 1 of 4 treatments: High fiber control (HF CON; basal diet control, corn silage + dried distillers’ grain, to meet protein requirements; n = 3), SARA control (SARA CON; n = 3), SARA + monensin treatment (MON CON; 200 mg d^-1^ monensin; n = 3), or SARA + biochanin A treatment (BCA; 6 g d^-1^ biochanin A; n = 3). Rumen fluid samples were taken at the end of the adaptation period (100% basal diet), high fiber period (100% basal diet + treatments), 60:40 SARA challenge period (60% basal diet + 40% cracked corn + treatments), and 30:70 SARA challenge period (30% basal diet + 70% cracked corn + treatments) for gelatin-hydrolyzing bacteria enumeration. The enumerations were performed in anaerobic liquid media with gelatin as the growth substrate. The tubes were incubated (39°C, 5 d), and the final dilution exhibiting growth (visual examination of gelatin hydrolysis after 1 h at 4°C) was recorded as the viable number. Means lacking a common English letter are different within sample day (*P* < 0.05). Means lacking a common Greek letter are different over sample days within treatment (*P* < 0.05). Treatment: *P* = 0.0023, sample day: *P* = 0.0031, and treatment × sample day: *P* = 0.1674; Pooled SEM: Treatment = 0.0680, sample day = 0.0970, treatment × sample day = 0.1939 (log transformed).
(TIF)
