# Supplementary figures and images for: Effect of biochanin A on the rumen microbial community of Holstein steers consuming a high fiber diet and subjected to a subacute acidosis challenge
Source: PLoS One. 2021 Jul 21;16(7):e0253754. doi: 10.1371/journal.pone.0253754 (PMC8294529; doi:10.1371/journal.pone.0253754)

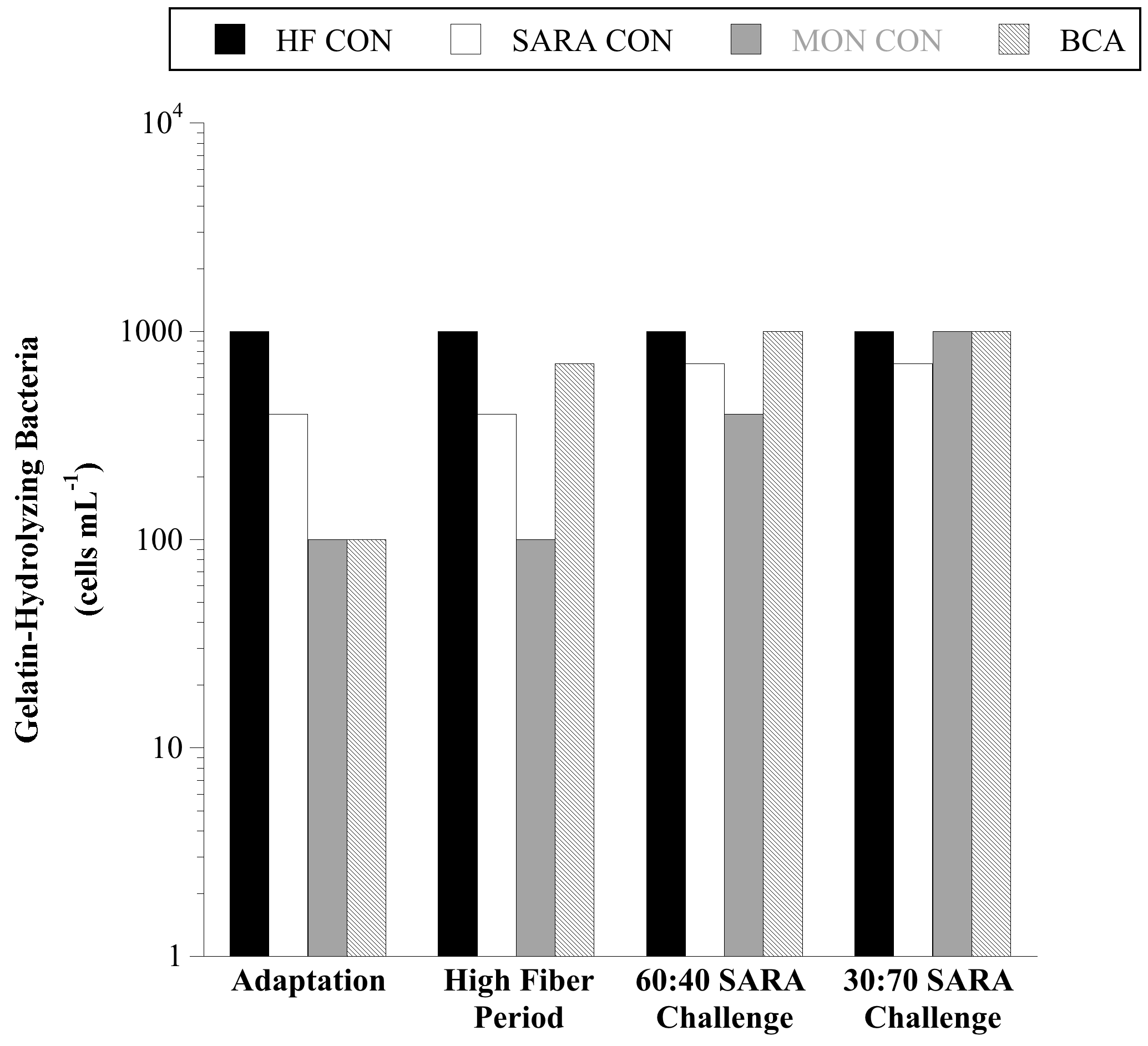

Supplement: S1 Fig — Holstein steers (n = 12) were blocked by weight into 1 of 4 treatments: High fiber control (HF CON; basal diet control, corn silage + dried distillers’ grain, to meet protein requirements; n = 3), SARA control (SARA CON; n = 3), SARA + monensin treatment (MON CON; 200 mg d-1 monensin; n = 3), or SARA + biochanin A treatment (BCA; 6 g d-1 biochanin A; n = 3). Rumen fluid samples were taken at the end of the adaptation period (100% basal diet), high fiber period (100% basal diet + treatments), 60:40 SARA challenge period (60% basal diet + 40% cracked corn + treatments), and 30:70 SARA challenge period (30% basal diet + 70% cracked corn + treatments) for gelatin-hydrolyzing bacteria enumeration. The enumerations were performed in anaerobic liquid media with gelatin as the growth substrate. The tubes were incubated (39°C, 5 d), and the final dilution exhibiting growth (visual examination of gelatin hydrolysis after 1 h at 4°C) was recorded as the viable number. Means lacking a common English letter are different within sample day (P < 0.05). Means lacking a common Greek letter are different over sample days within treatment (P < 0.05). Treatment: P = 0.0023, sample day: P = 0.0031, and treatment × sample day: P = 0.1674; Pooled SEM: Treatment = 0.0680, sample day = 0.0970, treatment × sample day = 0.1939 (log transformed). (TIF) [file pone.0253754.s001.tif]
